# Smokeless tobacco use and risk of oral cavity cancer

**DOI:** 10.3906/sag-1809-11

**Published:** 2020-04-09

**Authors:** Shah Zeb KHAN, Ahmed FAROOQ, Misbah MASOOD, Abubaker SHAHID, Irfan Ullah KHAN, Hasan NISAR, Ismat FATIMA

**Affiliations:** 1 Institute of Nuclear Medicine and Oncology (INMOL), Lahore Pakistan2; 2 Faculty of Medicine, Pakistan Institute of Engineering and Applied Sciences (PIEAS), Nilore, Islamabad Pakistan

**Keywords:** Smokeless tobacco, risk, snuff, oral cavity

## Abstract

**Background/aim:**

Smokeless tobacco has been associated with oral cavity cancer for several decades. The incidence of oral cavity cancer is higher in some parts of the world especially South and South-East Asia including Pakistan. The aim of current study was to evaluate the risk of oral cavity cancer among smokeless tobacco users in our country.

**Materials and methods:**

A case-control study was conducted between November 2016 and September 2017. Patients diagnosed with oral cavity cancer receiving treatment were included as cases and the attendants of various cancer patients visiting the hospital during the study period were included in the study as controls. Odds ratios (OR) and 95% confidence intervals (CI) were calculated and all reported P-values were considered significant at < 0.05.

**Results:**

The crude OR for the “ever smokeless tobacco users” among cases and controls came out to be 4.98 (95%CI; 2.76–9.01). The OR for snuff users among cases and controls was 4.82 (95%CI; 2.37–9.80) and that for betel leaf users was 4.42 (95%CI; 1.66–11.91) after adjusting for smoking and age.

**Conclusion:**

Our study provided strong evidence for snuff and betel leaf to be independent risk factors for oral cavity cancer.

## 1. Introduction

International Agency for Research on Cancer (IARC) in 2006 declared that smokeless tobacco (SLT) is carcinogenic in human beings, causing cancer of the oral cavity and pancreas. Wide variability amongst geographic regions in the type and extent of disease caused by the use of smokeless tobacco was observed by IARC and disease dissimilarities were attributed to the large differences in the concentrations of carcinogens in the tobacco used in different regions [1–5].

Smokeless tobacco has capricious modes of consumption from chewable tobacco not mixed with any other ingredient to a mixture of tobacco with other ingredients such as betel leaf (locally called “paan”), snuff (locally called “naswar”), supari, chalia, and Mishri [6]. It contains a number of carcinogens including non-volatile alkaloid-derived tobacco-specific N-nitrosamine and N-nitrosamino acids. In addition, some other carcinogens such as volatile aldehydes, and some poly-nuclear agents have also been identified in SLT [7].

The most prevalent form of the oral cavity cancer in South and South East Asia is squamous cell carcinoma (approximately 90%) because of cultural use of betel-leaf and different forms of smokeless tobacco while the other forms are adenocarcinoma of the salivary glands, malignant melanoma, and adenoid cystic carcinoma. Pakistan is one of the countries where the use of SLT is a culturally acceptable habit. Oral cavity cancer has become the most common cancer among men and the second most common cancer among both sexes in Pakistan [8]. An estimated 6000 Pakistanis lose their lives because of oral cavity cancer every year [9]. Studies from Karachi demonstrate that snuff is a major contributor in the aetiology of oral cavity cancer in Pakistan [8].

We conducted a hospital based case-control study with the aim to further evaluate the risk of oral cavity cancer by the use of various smokeless tobacco products. Rationale of the study was that there has been no case-control study done in the past in our region to show direct association of smokeless tobacco use and risk of oral cavity cancer. Moreover, most common smokeless tobacco consumed in our region is snuff in contrast to the other regions where betel leaf, supari, and chalia are more common.

## 2. Materials and methods

A case-control study was conducted at INMOL Hospital, Lahore between November 2016 and September 2017. Patients diagnosed with oral cavity cancer receiving treatment at INMOL Hospital were included in the study as cases and the attendants of various cancer patients visiting the hospital during the study period were selected as controls. A case was defined as a person aged 19 years and above with laboratory confirmed primary diagnosis of oral cavity cancer. All cases that were histopathologically diagnosed as oral cavity cancer on or after January 1, 2016 and visited the hospital during the study period were included. Cases diagnosed before the beginning of 2016, metastatic lesions in the oral cavity from other sites and tumours of major salivary glands were excluded from our study. People with history of diseases or conditions which could have causal association with oral cavity cancer were also excluded to limit confounding factors. This included alcohol consumption, uncontrolled diabetes, poor fitting dentures, and Lichen Planus. To ensure relative ethnic and socioeconomic similarity with cases, a control was defined as a person aged 19 years and above not having oral cavity cancer, who visited INMOL as an attendant of a case selected for our study. Ethical approval of the study was obtained from Institutional Review Board and informed verbal consent was taken from the participants.

### 2.1. Data collection

Sample size consisted of 90 cases and 120 controls. The cases and controls were personally interviewed using a structured questionnaire. The questionnaire included questions regarding demographic information such as age, gender, literacy level, province, and subsite of tumour. The second part of the questionnaire aimed at questions on the use of smokeless tobacco, which comprised questions on type, frequency, and duration of the habits. “Ever smokeless tobacco users” were defined as those who had used smokeless tobacco at least 20 times in their life. Age was categorized into 15-year bands from 19–60 years and participants above 60 years of age were categorized separately.

### 2.2. Statistical methods

Frequencies with percentages were used for categorical variables. Chi square test was used for calculating P-values in categorical variables. All reported P-values were calculated with significance considered at P < 0.05. Crude (unadjusted) Odds ratios (OR) with 95% confidence interval (95%CI) were calculated and then adjusted for potential confounders, i.e. age, gender, and smoking using multinomial logistic regression analysis. Never SLT users, male gender, age of >50 years, and never smokers served as reference category in logistic regression analysis. Statistical analysis was performed using SPSS version 20.0 (IBM Corp., Armonk, NY, USA). Tumour sites were categorized according to the International Classification of Diseases, Tenth Revision (ICD-10). The staging system used for oral cavity cancer was TNM classification system 2010, 7th edition, maintained by The American Joint Committee on Cancer (AJCC) and the International Union for Cancer Control (UICC).

## 3. Results

### 3.1. Sample characteristics

Mean age of the controls was 52.04 years (Range: 22–69, ± SD 11.17) and for cases it was 57.38 years (Range: 40–70, ± SD 6.88).Majority of study participants were in the age group between 46–65 years (Table 1). Cases and controls were age matched (P = 0.090), (Table 1). Of the 90 cases, 62.2% were males and 37.8% were females (P < 0.001) as shown in Table 1. Male to female ratio was 1.65:1. Of the 120 controls, the males and females were 88.3% and 11.7%, respectively (P <0.001). In our control group male to female ratio was 7.5:1. The major participants in cases and controls were from the provinces of Punjab and KPK (e.g., among cases 46.2% and 40.0%, respectively). Also, there were 6.7% of cases from Gilgit Baltistan and Azad Kashmir. No geographical differences occurred between cases and controls (P = 0.625).The large proportion of participants was illiterate both in cases and controls (62.2% vs. 56.7%). 17.8% of cases had completed primary level of education and 4.4% had completed graduation (so the questionnaire used during data collection was designed in their local languages as lack of understanding of English or Urdu among participants, especially among KPK females, was common).Smoking status was similar in both cases and controls (0.134). Use of SLT products was significantly high in cases as compared to controls (P <0.001). Out of 90 cases, 58 (64.4%) had the habit of SLT use while out of 120 controls, 32 (26.7%) had this habit (Table 1).

**Table 1:** Baseline characteristics of cases and controls.

Age (years)	Cases	Controls	P-values
19–30	0	10 (8.3%)	0.090
31–45	8 (8.9%)	18 (15%)	
46-60	58 (64.4%)	66 (55%)	
>60	24 (26.7%)	26 (21.7%)	
Gender			
Male	56 (62.2%)	106 (88.3%)	<0.001
Female	34 (37.8%)	14 (11.7%)	
Province				Federal	6 (6.7%)	14 (11.7%)	0.625
Punjab	42 (46.7%)	55 (45.8%)					
KPK	36 (40%)	42 (35%)					
Baluchistan	0	0					
GilgitBaltistan/Azad Kashmir	6 (6.7%)	9 (7.5%)					
Sindh	0	0					
Literacy			
Primary	16 (17.8%)	30 (25.0%)	0.003
Middle	0	10 (8.3%)	
High / Intermediate	14 (15.5%)	12 (10.0%)	
Graduate	4 (4.4%)	0	
Illiterate	56 (62.2%)	68 (56.7%)	
Smoking status:				Ever smokers	18 (20%)	16 (13%)	0.134
SLT users	58(64.4%)	32(26.7%)	<0.001

#### 3.1.1. Tumour subtypes and staging

**Figure 1:**
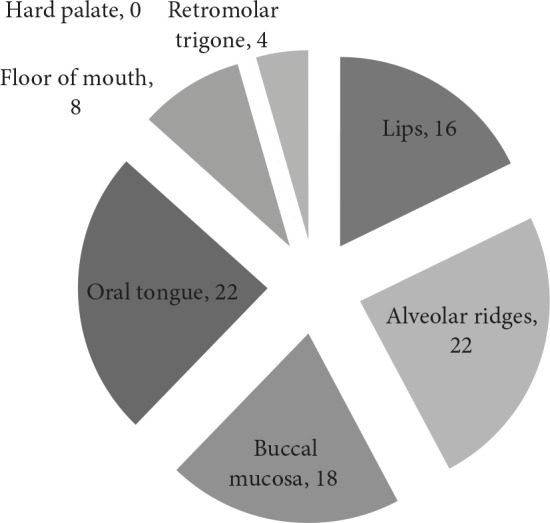
Frequency Distribution of cases by their tumour subsites.

Oral tongue carcinoma and alveolar ridge (upper or lower) carcinoma were detected in 24.4% of cases followed by buccal mucosa cancer in 20.0% of cases. Rest of the subsites included floor of mouth in 8.9% of cases and lips in 17.7% of cases. Only 4.4% of cases were having cancer of Retro molar trigone and there was no case of hard palate cancer (Figure 1).

The Tumour size (T) according to AJCC TNM (7th edition) staging was classified as T4 in 40.0% cases and T3 in 35.5% cases. The nodal involvement (N) was seen in most cases with 42.2% having N1 disease and 31.1% having N2 disease. It was interesting to see that almost all the cases were not having distant metastases (Table 2).

**Table 2:** Frequency distribution of cases by their TNM stage.

Tumour Size (T)	No. of Cases
T1	10 (11.1%)
T2	22 (24.4%)
T3	32 (35.5%)
T4	36 (40.0%)
Node status (N)	
N0	10 (11.1%)
N1	38 (42.2%)
N2	28 (31.1%)
N3	14 (15.5%)
Distant mets (M)	
M0	90 (100%)
M1	0
Grade	
G I (well-differentiated)	10 (11.1%)
G II (moderately differentiated)	22 (24.4%)
G III (poorly differentiated)	30 (33.3%)
G IV (undifferentiated)	12 (13.3%)
Missing data	16 (17.8%)
Anatomic stage	
Stage I	0
Stage II	12 (13.3%)
Stage III	38 (42.2%)
Stage IV	40 (44.4%)

### 3.2. Smokeless tobacco users

**Figure 2:**
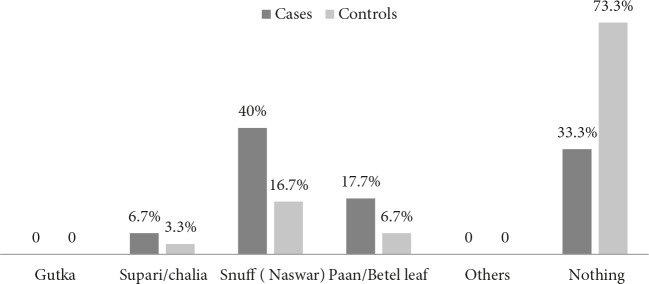
Smokeless tobacco users among cases and controls.

In our study, 40.0% of cases consumed snuff compared to 16.7% of controls; 6.7% of cases used supari/chalia compared to 3.3% of controls;1 7.7% of cases consumed betel leaf compared to 6.7% of controls (Figure2).

### 3.3. OR calculation

The OR was calculated for the ever smokeless tobacco (SLT) users among cases and controls; it came out to be 4.98 (95%CI; 2.76–9.01) with p-value of <0.0001 as shown in Table 3. After adjusting for age and smoking the adjusted OR came out to be 4.71 (95%CI; 2.53–8.74) which was again highly significant (P < 0.001). After adjusting for gender, females had highly significant (P < 0.001) many-fold increased risk of oral cavity cancer as compared to men in our study participants and the OR value increased up to 28.29 (95%CI; 9.93–80.52), (Table 3).

**Table 3:** Association of oral cavity cancer with smokeless tobacco (SLT) use in study subjects.

Variables	Cases(n = 90)	Controls(n = 120)	Unadjusted OR	95% CI	Adjusted ORa	95% CIa	Adjusted ORb	95% CIb
SLT users	58	32	4.98	2.76–9.01	4.71	2.53–8.74	28.29	9.93–80.52
Never user	32	88	1.00 (referent)	-	1.00 (referent)	-	1.00 (referent)	
Type of SLT used:								
Snuff	36	20	4.95	2.51–9.77	4.82	2.37–9.80	32.65	10.6–100.4
Betel leaf	16	8	5.50	2.15–14.08	4.42	1.66–11.91	23.18	6.23–86.2
Supari/chalia	6	4	4.12	1.09–15.57	4.67	1.14–19.12	21.09	3.59–123.6
Never users	32	88	1.00 (referent)	-	1.00 (referent)	-	1.00 (referent)	-
Duration of snuff use:								
<10 years	10	5	3.70	1.20–11.4	5.45	1.59–18.71	21.44	4.89–94.01
10–20 years	4	6	1.23	0.33–4.56	1.73	0.43–6.99	5.81	1.23–27.45
>20 years	22	9	4.52	1.95–10.52	3.25	1.37–7.71	6.45	2.50–16.65
Never users	54	100	1.00 (referent)	-	1.00 (referent)	-	1.00 (referent)	-
Frequency of snuff use:								
Daily			5.22	2.66–10.27	5.22	2.56–10.65	34.5	11.2–106.1
Frequently in a week			6.60	2.16–20.2	6.80	2.09–22.1	45.8	9.74–216.1
Weekly			5.50	0.961–31.5	4.82	0.76–30.4	32.4	3.64–287.8
Frequently in a month			2.20	0.56–8.71	1.43	0.35–5.83	5.48	0.97–31.03
No			1.00 (referent)	-	1.00 (referent)	-	1.00 (referent)	-

a: OR adjusted for age (≤50 and >50 years) and smoking (never vs. ever smokers). b: OR adjusted for age (≤50 and >50 years), gender, and smoking (never vs. ever smokers).

The adjusted OR of snuff users was the highest being 4.82 (95%CI; 2.37–9.80) with P-value of <0.001 when adjusted for age and smoking. For supari/chalia users, the OR was 4.67 (95%CI; 1.14–19.12) with P-value of 0.032. For betel leaf users, the OR was 4.42 (95%CI; 1.66–11.91) with P-value of 0.003 as shown in Table 3.

Subjects with snuff and betel leaf use were significantly associated with oral cavity risk after adjustment with age, gender, and smoking (Table 3).

### 3.4. Tumour characteristics among snuff users

The most common subsites of oral cavity cancer found in these participants were alveolar ridge (50%) and buccal mucosa (28%). The T3 size (39%) and N2 (42%) node stage of the tumour was found to be more prevalent among snuff users and the patients were found in maximum numbers with Stage III disease (47%).

#### 3.4.1. Snuff use and its duration

Snuff users were divided into 3 groups based on their duration of snuff use, i.e. less than 10 years, 10–20 years and more than 20 years. These groups were then compared separately with never users of any smokeless tobacco products. The OR was found to be higher than 1 in all groups with the highest being in those with more than 20 years history of snuff use (OR = 4.52 and P-value < 0.001), (Table 3). Daily and weekly users were more prone to oral cavity cancer while infrequent users had no significant association with oral cavity cancer (P = 0.261) despite an OR of 2.20 (Table 3).

**Figure 3:**
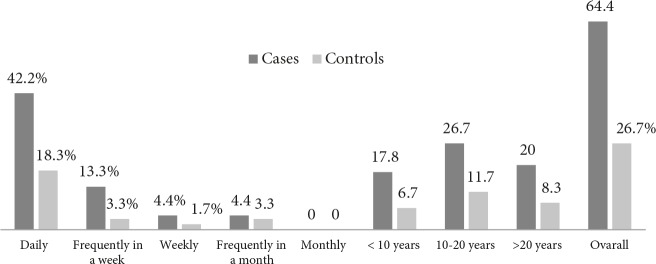
Frequency and duration of smokeless tobacco use among cases and controls expressed in percentages.

Frequency and duration of smokeless tobacco use among cases and controls are expressed in Figure 3 while frequency and duration of SLT use were prevalent among cases as compared to the control subjects (Figure 3).

## 4. Discussion

The use of SLT is common among different South Asian countries like Pakistan and India. Commonly available smokeless tobacco products in Pakistan include snuff and betel leaf while less commonly available ones include gutka, supari/chalia, and some chewing tobacco.

Present study was intended as a pilot project to evaluate the risk of oral cavity cancer in Pakistani population using smokeless tobacco products. We have observed a positive association between use of smokeless tobacco and oral cavity cancer among participants. OR for the ever smokeless tobacco users was calculated. The participants who consumed smokeless tobacco were approximately 5 times (OR = 4.98) more likely to get oral cavity cancer compared to never smokeless tobacco users. This value was statistically significant with P-value of 0.0001. Such a marked and statistically significant increase in OR warrants the need for a future study with a much larger sample size to ensure adequate study power.

Our findings are consistent with studies conducted in other countries of the region reporting the oral cavity cancer specific relative risk ranging from 1.2–12.9 with the use of SLT. The pooled OR for chewing tobacco and risk of oral cancer has been calculated as 4.7(3.1–7.1) in a recent meta-analysis comparing the studies in South Asia [9–11]. However, some differences associated with risk estimates were observed in the populations predominantly using Gutka and Betel-quid or snuff as a medium. The enormity of risk for oral cancer is linked with the use of snuff in comparison with other SLT products. The estimated OR for snuff users vs. never users rises up to 23.7(6.9–81.0) [10].

Studies from Europe and North America have reported the relative risk of 1.8 (1.1-2.9) for developing oral cancer by using SLT products [12]. Regional disparity among studies for relative risk of developing oral cancer may reflect the variance of SLT products used in different regions. Snuff contributes to about 40% of oral cancers in the study region. The participants of our study who consumed supari/chalia were having adjusted for age and smoking, OR of 4.67 (statistically significant with P-value of 0.032). For betel users, the OR showed 4.4 times (OR = 4.42) more likelihood of getting oral cavity cancer in comparison to nonusers. The participants who were snuff users had 4.82 times greater likelihood of developing oral cavity cancer as compared to nonusers (P < 0.001), (Table 4). Use of SLT products in females increases the likelihood of oral cavity cancer up to 28.29 times (9.93–80.52), Table 3. This may be due to a reduced basal risk of oral malignancy among Pakistani women due to lower prevalence of alcohol consumption and smoking among them, 2 other major risk factors for developing oral cancer. It is also in line with South Asian studies where OR among women ranged between 6.5–45.8 in women in contrast to men having values between 1.5–10.9 [10]. Keeping in view the many fold increase in risk of oral cavity cancer among women, we have adjusted ORs with age and smoking separately and age, gender, and smoking in the last column of Table 3 to outweigh the effect of gender in the cancer risk (Table 3). Duration and frequency of snuff use also had impact on OR of oral cavity cancer risk which is also in concordance with other South Asian studies [10].

Our study showed that the most common risk factor for oral cavity cancer was snuff and common sub-site in cases that consumed snuff was buccal mucosa and alveolar ridge (upper/lower). The literature issued by IARC on smokeless tobacco use showed that maximum number of cases were reported of buccal mucosa cancer in snuff users as these regions of oral cavity come in direct contact of the snuff [5]. Also a meta-analysis published on smokeless tobacco use among head and neck cancer cases showed that snuff use was more frequently associated with buccal mucosa cancer [7].

Similar to the other case-control studies, one of the main limitations of the study was recall-bias and selection-bias to some extent. To minimize recall-bias, the cases diagnosed on and after January 1, 2016 were selected. The proportion of females in the control group was less. The probable reason could be lack of privacy in a hospital setting. Another limitation is that the subjects were derived from a hospital and therefore, may not approximate the relative risk for the general population. Furthermore, large studies are needed which should examine smokeless tobacco use separately from joint smoking and smokeless tobacco use to predict its association with oral cavity cancer among both types of users.

In spite of the limitations, our study came out with the conclusion that the use of smokeless tobacco results in exposure to potent carcinogens. Our studies showed a positive association between ever use of smokeless tobacco and risk of oral cavity cancer. Snuff was consumed by maximum participants in our study. It showed association with increase in duration of snuff use as more than 20 year-users have more likelihood of getting oral cavity cancer than never users of snuff. Also, the use of betel showed positive association with oral cavity cancer risk. 

In conclusion, our study showed a statistically significant positive association between use of smokeless tobacco and risk of oral cancer in patients presenting to our Institute from the north and centre of Pakistan, even more so in women. Literature review shows that this is in line with similar studies carried out in the region and other parts of the world. A higher powered study with sampling from other parts of the country could help in better understanding of disease epidemiology as well as has a greater impact on public awareness.

## Acknowledgement/Disclaimers/Conflict of Interest

The manuscript contains original data from patient’s population, visiting to INMOL for diagnosis and treatment of cancer which have not been previously published and have not been submitted for publication elsewhere. It is furthermore declared that all authors have contributed significantly in the research and also in preparation of the manuscript. Finally, all authors have read the manuscript and agreed with the submission with no conflicts of interest to disclose.

## Informed Consent

The data included in this research were collected from patients after getting their consent.
